# Phosphate Transport Through Homogeneous and Heterogeneous Anion-Exchange Membranes: A Chronopotentiometric Study for Electrodialytic Applications

**DOI:** 10.3390/membranes15080230

**Published:** 2025-07-31

**Authors:** Kayo Santana-Barros, Manuel César Martí-Calatayud, Svetlozar Velizarov, Valentín Pérez-Herranz

**Affiliations:** 1 IEC Group, Research Institute for Industrial, Radiophysical and Environmental Safety (ISIRYM), Universitat Politècnica de València, Camí de Vera s/n, 46022, P.O. Box 22012, E-46071 València, Spain; vperez@iqn.upv.es; 2 LAQV/REQUIMTE, Department of Chemistry, NOVA School of Science and Technology, NOVA FCT, Universidade NOVA de Lisboa, 2829-516 Caparica, Portugal; s.velizarov@fct.unl.pt

**Keywords:** nutrient recovery, phosphorus, electrodialysis, membrane potential drop, electroconvection, water dissociation

## Abstract

This study investigates the behavior of phosphate ion transport through two structurally distinct anion-exchange membranes—AMV (homogeneous) and HC-A (heterogeneous)—in an electrodialysis system under both static and stirred conditions at varying pH levels. Chronopotentiometric and current–voltage analyses were used to investigate the influence of pH and hydrodynamics on ion transport. Under underlimiting (ohmic) conditions, the AMV membrane exhibited simultaneous transport of H_2_PO_4_^−^ and HPO_4_^2−^ ions at neutral and mildly alkaline pH, while such behavior was not verified at acidic pH and in all cases for the HC-A membrane. Under overlimiting current conditions, AMV favored electroconvection at low pH and exhibited significant water dissociation at high pH, leading to local pH shifts and chemical equilibrium displacement at the membrane–solution interface. In contrast, the HC-A membrane operated predominantly under strong electroconvective regimes, regardless of the pH value, without evidence of water dissociation or equilibrium change phenomena. Stirring significantly impacted the electrochemical responses: it altered the chronopotentiogram profiles through the emergence of intense oscillations in membrane potential drop at overlimiting currents and modified the current–voltage behavior by increasing the limiting current density, reducing electrical resistance, and compressing the plateau region that separates ohmic and overlimiting regimes. Additionally, both membranes showed signs of NH_3_ formation at the anodic-side interface under pH 7–8, associated with increased electrical resistance. These findings reveal distinct ionic transport characteristics and hydrodynamic sensitivities of the membranes, thus providing valuable insights for optimizing phosphate recovery via electrodialysis.

## 1. Introduction

Phosphorus recovery has gained increasing attention due to its vital role in agriculture. The growing global population demands higher food production, which in turn drives the need for fertilizers containing phosphorus [1]. Consequently, phosphate rock has been designated as a critical raw material by the European Union in recent years [2]. Conversely, excessive phosphate release into the environment from municipal wastewater, animal waste, and landfill leachates contributes to the eutrophication of aquatic ecosystems, posing serious environmental risks [3]. A clear example of the challenges posed by nutrient pollution in aquatic ecosystems can be seen in *L’Albufera* Lake, located in València, Spain. This coastal lagoon has long been surrounded by rice paddies, and over time, the intensification of agricultural activity has contributed to rising concentrations of nitrogen and phosphorus in its waters. The combination of continuous nutrient input and limited freshwater renewal has progressively altered the lake’s ecological dynamics. Recent investigations have provided a detailed picture of these transformations, revealing how shifts in water chemistry are affecting both ecosystem health and biodiversity [4,5]. This dual challenge—the necessity of securing phosphate resources to ensure global food security, alongside the imperative to remove phosphate from water bodies to mitigate eutrophication—has motivated extensive research on developing efficient extraction and recovery processes for phosphorus. Among these, electrodialysis has emerged as a promising technology, valued for its versatility and its ability to operate without requiring the addition of chemical reagents [6,7].

A conventional electrodialysis system comprises ion-exchange membranes that selectively transport anions (anion-exchange membranes, AEMs) or cations (cation-exchange membranes, CEMs). Ion transport rates are generally governed by the applied current, membrane properties, and solution characteristics. However, when dealing with phosphoric acid (H_3_PO_4_), which is a weak electrolyte, ion transport is influenced by an additional factor not relevant in systems with strong electrolytes such as NaCl. Phosphate speciation and transport are highly dependent on the solution pH due to equilibrium shifts involving protonation/deprotonation reactions, leading to the formation of distinct species—H_3_PO_4_, H_2_PO_4_^−^, HPO_4_^2−^, or PO_4_^3−^—that predominate at different pH levels, which may influence the process [8].

In electrodialysis systems, this phenomenon of equilibrium shifts is especially significant near the membrane surface, where water dissociation may occur, inducing local pH changes, altering the chemical equilibrium and consequently the phosphate species available for transport [9]. Therefore, understanding the mechanisms governing the transport of weak electrolytes like phosphate through ion-exchange membranes is crucial to optimize electrodialysis performance. Chronopotentiometry has been widely employed to investigate these mechanisms, particularly to elucidate how equilibrium shifts affect phosphate ion transport. Previous studies have explored the effects of membrane heterogeneity [10], type of membrane functional group [9,11], and solution conditions such as ionic concentration and pH [12] on the process performance. Additionally, several modeling efforts have aimed to predict phosphate ion transport behavior under controlled conditions [8,13].

Despite the considerable progress made in understanding ion transport phenomena in electrodialysis systems, a large proportion of studies to date have been performed under stagnant (without forced convection) conditions, where the solution remains unstirred. However, practical electrodialysis operations typically involve solution flow and turbulence, primarily due to the circulation of the electrolyte and the presence of spacers that promote mixing and reduce concentration polarization [14,15]. This hydrodynamic environment can profoundly influence ion transport behavior, including the magnitude of the limiting current density, overlimiting transport mechanisms, and membrane fouling [16,17]. Nevertheless, the effects of flow conditions and hydrodynamic forces on ion migration—particularly for weak electrolytes—require further investigation.

In an effort to address this knowledge gap, the present study focuses on the transport of phosphate ions through commercial AEMs. Chronopotentiometry was employed as a technique to investigate the ion transport mechanisms in the presence and absence of solution agitation. Two types of AEMs were examined: a homogeneous membrane (AMV) and a heterogeneous membrane (HC-A). Experiments were conducted using 0.05 mol/L ammonium hydrogen phosphate ((NH_4_)_2_HPO_4_) solutions at different pH values (4.4, 7, and 8), and under both stagnant and stirred conditions, simulating distinct hydrodynamic situations.

This comparative approach enables the evaluation of how membrane structure and hydrodynamic conditions affect the electrochemical response and transport efficiency of phosphate ions. The findings are expected to offer valuable insights into the optimization of membrane performance for phosphorus recovery, contributing to the development of more efficient and scalable electrodialysis systems aligned with circular economy principles and sustainable nutrient management.

## 2. Materials and Methods

### 2.1. Electrochemical Cell

**Figure 1:**
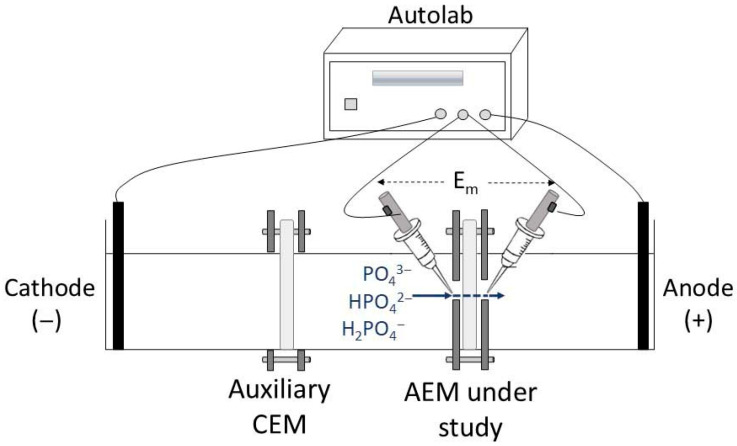
Representation of the electrochemical cell used.

Figure 1 and Figure S1 of the Supplementary Material present a schematic representation and a photograph of the electrochemical cell used, respectively. The cell consisted of three compartments with an AEM and a CEM separating the anode and cathode chambers, respectively, from the central (dilute) compartment. The three compartments contained the same working solution in each experiment. The membranes under evaluation (AEM) had an effective area of 1 cm^2^, while the effective area of the auxiliary CEM membrane was 12.6 cm^2^. The latter was included to minimize the effects of cathodic reactions on the ionic transport through the AEM, by restricting hydroxyl ions transport toward the central compartment. Two graphite electrodes were present at each end of the cell for the application of electric current, which were connected to an Autolab PGSTAT302N potentiostat/galvanostat (Metrohm, Herisau, Switzerland). Ag/AgCl reference electrodes, immersed in Luggin capillaries, were placed at each surface of the membrane under evaluation for measuring the potential drop (E_m_) of the membrane/solution system under different current densities. The experiments were conducted at room-controlled temperature (approximately 25 °C) both with and without stirring. In the experiments with stirring, only the central compartment was agitated using a magnetic stirrer at 400 rpm, ensuring solution homogenization without vortex formation. Prior to each experiment, the membranes were kept immersed in the working solution for 24 h to reach an equilibrium of counter-ions exchanged with the membrane fixed charges.

### 2.2. Obtention of Chronopotentiometric and Polarization Curves

First, linear sweep voltammetry (LSV) curves were registered for each membrane/solution system evaluated, applying a scan rate of 2 mV/s. In each evaluation, at least 3 scans were performed to ensure that the current–voltage behavior of the membrane/solution system was stable. Next, the solution was renewed. Then, chronopotentiometric curves (ChPs) were constructed according to the following steps: (1) measurement of E_m_ for 10 s without applying current to stabilize the system, (2) measurement of E_m_ for 300 s under the application of an electrical current, and (3) measurement of E_m_ for 100 s without applying current to allow for the relaxation of the membrane/solution system. The current values applied in step (2) were increased and defined based on the LSV, such that approximately 5 values corresponded to the ohmic region, 5 to the plateau region, and 5 to the overlimiting region.

Current–voltage curves (CVCs) were represented by plotting the applied current density vs. the E_m_ values measured at the end of step (2) of the chronopotentiograms, immediately before the current application was interrupted. From the obtained CVCs, the limiting current density (*i_lim_*) and the electric resistance when operating at ohmic (*R_ohm_*) and overlimiting (*R_overlim_*) conditions were determined. *i_lim_* was determined using the Cowan–Brown method, by plotting the membrane potential drop/current density ratio (E_m_*/i*) as a function of the inverse of current density (1/*i*) [18]. *R_ohm_* and *R_overlim_* were calculated by determining the inverse of the slope of the tangent of the first (ohmic) and third (overlimiting) regions, respectively. A detailed description of the procedure followed can be found in [19]. The experiments were conducted in duplicate, and the estimated relative errors between the results were below 5%.

### 2.3. Ion-Exchange Membranes

Two AEMs were evaluated, namely, Selemion AMV (Asahi Glass Co, Tokyo, Japan) and Ionsep-HC-A (Hangzhou Iontech Environmental Technology Co., Ltd., Hangzhou, China), also known in the literature as HDX200. Both AEMs contain quaternary ammonium groups as fixed charges, with their main differences being heterogeneity and thickness. These membranes were selected for testing because they exhibit typical characteristics of commercial homogeneous and heterogeneous membranes, respectively. A CEM Nafion 117 (DuPont, Wilmington, DE, USA) was also present in the electrochemical cell as an auxiliary membrane. This membrane contains sulfonic acid groups as fixed charges and is homogeneous. The key characteristics of the membranes are shown in Table 1.

### 2.4. Working Solutions

The working solutions were prepared with deionized water and diammonium phosphate (NH_4_)_2_HPO_4_ (Sigma Aldrich, Burlington, MA, USA) at a concentration of 0.05 mol/L and pH values of 4.4, 7, and 8. Analytical grade phosphoric acid (H_3_PO_4_) (Sigma Aldrich, Burlington, MA, USA) was used for pH adjustment.

## 3. Results

### 3.1. Speciation Diagram

**Figure 2:**
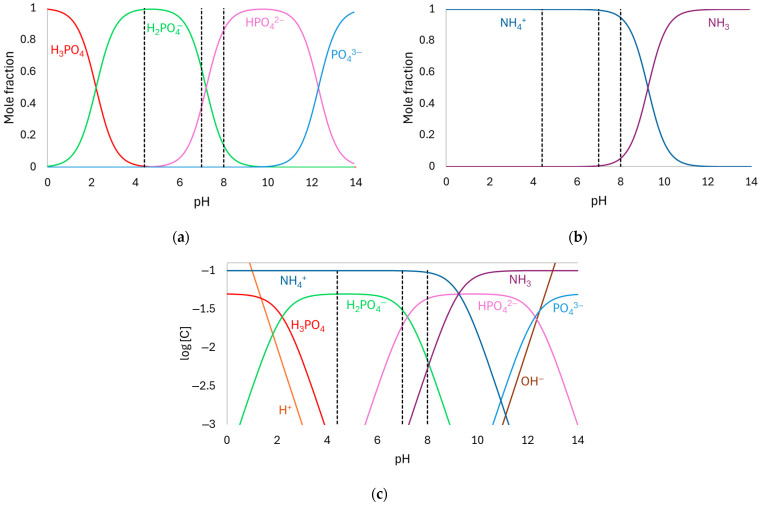
Speciation diagram of (**a**) phosphate, (**b**) ammonium, and (**c**) phosphate and ammonium compounds in aqueous solution. The dashed lines indicate the specific pH values examined in this study (4.4, 7, and 8).

The phosphate speciation diagram (mole fraction vs. pH) presented in Figure 2a was constructed based on the equilibrium represented by Equations (1)–(3) [12]. As observed, H_2_PO_4_^−^ is the predominant species at pH 4.4. At pH 7, both H_2_PO_4_^−^ and HPO_4_^2−^ are present at comparable concentrations, while at pH 8, HPO_4_^2−^ becomes the dominant species. All these species are expected to be transported across the AEM.

(1) $$H_{3}{PO}_{4}⇄H_{2}PO_{4}^{-}+H^{+}\;\;\;\;\;{pK}_{a}=2.2$$

(2) $$H_{2}PO_{4}^{-}⇄HPO_{4}^{2-}+H^{+}\;\;\;\;\;{pK}_{a}=7.2$$

(3) $$\;\;\;HPO_{4}^{2-}⇄PO_{4}^{3-}+H^{+}\;\;\;\;\;{pK}_{a}=12.3$$

Figure 2b shows the ammonium speciation diagram (mole fraction vs. pH) constructed using the equilibrium reaction shown in Equation (4). Within the pH range of the tested solutions (4.4–8), the predominant species is NH_4_^+^, which is expected to migrate through the CEM [28].

(4) $$NH_{4}^{+}↔{NH}_{3}+H^{+}\;\;\;\;\;{pK}_{a}=9.25$$

Using the mole fraction data shown in Figure 2a,b, the speciation diagram of all species present in the solution was constructed (log [C] vs. pH), as presented in Figure 2c. From the molar concentrations of the anionic species *j* shown in the figure (*C_j_*) and their valency (*z_j_*), the equivalent anionic charge (*Q*_*eq*_^−^) of the solutions at pH 4.4, 7, and 8 was calculated using Equation (5), resulting in values of 49.8 meq/L, 69.3 meq/L, and 93.2 meq/L, respectively.

(5) $$Q_{eq}^{-}=\sum \left|z_{j}\right|C_{j}$$

#### 3.1.1. Stability of the Electrochemical Response of the Membrane/Solution System

The three voltammograms obtained with the AMV membrane and the 0.05 mol/L solution at pH 7 under stirring are presented in Figure S2 of the Supplementary Material. As shown in the figure, the system exhibited stable behavior from the second scan onward. Therefore, all subsequent curves refer to the second scan.

#### 3.1.2. Effect of Stirring on the Current–Voltage Response

**Figure 3:**
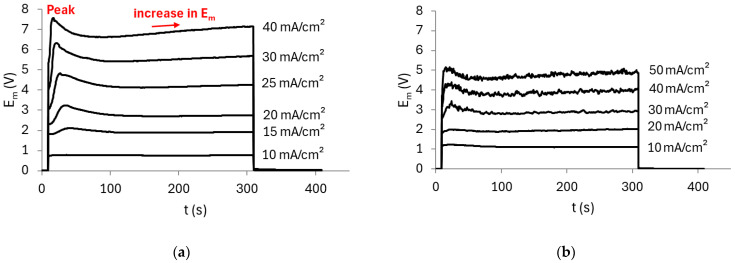
Chronopotentiometric curves obtained with the AMV membrane in a 0.05 mol/L solution at pH 7 (**a**) without stirring and (**b**) with stirring.

ChPs were recorded using the AMV membrane and a pH 7 solution, both with and without stirring, to evaluate the effect of forced convection on ion transport. Figure 3 shows the ChPs obtained.

In both cases, the curves obtained at the lowest current densities (e.g., 10 mA/cm^2^) remained constant over time, which is attributed to ion transport being governed by diffusion and mainly migration mechanisms across the membrane. As the applied current increased, the curves exhibited a rapid rise in E_m_ within a very short time after current application, reaching a peak (indicated in Figure 3a), followed by a drop in E_m_ until a quasi-steady state was achieved. It is well known that when the membrane/solution system operates above its limiting current density, the dominant transport mechanisms become electroconvection and/or gravitational convection, in addition to possible water dissociation taking place at the membrane surface. In the present case, the E_m_ peak likely emerged as a result of water dissociation occurring in the diffusion boundary layer, which induced an equilibrium shift in the membrane/solution system and consequently altered the conductivity of the species transported through the membrane [29,30], as will be discussed below. It is worth noting that the time required for the sharp increase in E_m_ (commonly referred to as the transition time) is shorter under stirred conditions, which can be explained by the enhancement of electroconvection due to forced convection [31]. Following the sharp drop in E_m_, a gradual increase over time is observed (also indicated in Figure 3a)—especially at higher current densities (e.g., 40 mA/cm^2^)—which can also be attributed to the shift in the chemical equilibrium occurring in both the diffusion boundary layer and within the membrane structure [12,32,33].

It is well known that AEMs catalyze the water dissociation reaction, which has a considerable impact on the transport behavior of weak electrolytes [15]. Here, during water dissociation, OH^−^ ions migrated across the membrane, while protons were retained at its cathodic/diluted surface due to the Donnan exclusion effect. This led to an increase in the internal pH of the membrane, while the pH at the cathodic side of the membrane decreased. As shown in the speciation diagram of phosphate compounds (Figure 2), at pH 7, H_2_PO_4_^−^ and HPO_4_^2−^ species are present at similar concentrations. However, at lower pH values, H_2_PO_4_^−^ becomes the predominant species, while from pH 7 onward, the predominant species is HPO_4_^2−^. In this case, the decrease in pH at the membrane surface led to the predominant uptake of H_2_PO_4_^−^ into the membrane, which has higher conductivity and therefore lowered the resistance of the cathodic/diluted surface of the membrane (diffusion coefficients of H_2_PO_4_^−^ and HPO_4_^2−^ are 0.959 × 10^−^^5^ cm^2^/s and 0.759 × 10^−^^5^ cm^2^/s, respectively [34]). However, once these ions entered the membrane, the high internal pH shifted the equilibrium again, resulting in the formation of HPO_4_^2−^ species, which became predominant at the anodic/concentrated surface of the membrane. These species exhibit lower conductivity, thereby increasing the resistance of the membrane, which explains the continuous increase in E_m_ observed in Figure 3. Similar behaviors have been observed by other authors when evaluating different anion-exchange membranes in phosphate-containing solutions [8,35,36].

The curves obtained under stirring conditions exhibited pronounced oscillations. It is worth noting that these oscillations were only observed at high current densities, exceeding the limiting current density of the membrane/solution system. According to Hernández-Pérez et al. [37], these intense oscillations can be attributed to enhanced solution mixing, which leads to a reduction in the thickness of the diffusion boundary layers. As a result, the solution delivered from the bulk to the diffusion boundary layer is significantly more concentrated than in the absence of stirring. This, in turn, generates greater concentration gradients, giving rise to a larger voltage oscillation amplitude. Additionally, the authors observed a direct relationship between forced convection and electroconvection in the ChPs that exhibited intense E_m_ oscillations. Comparing the curves from both figures at the same current density (e.g., 40 mA/cm^2^), it is evident that the E_m_ values measured without stirring are significantly higher than with stirring. This can be attributed to enhanced phosphate ion transport across the membrane system under stirring conditions, which led to a reduction in the membrane/solution system resistance, as will be further discussed in the CVCs below.

**Figure 4:**
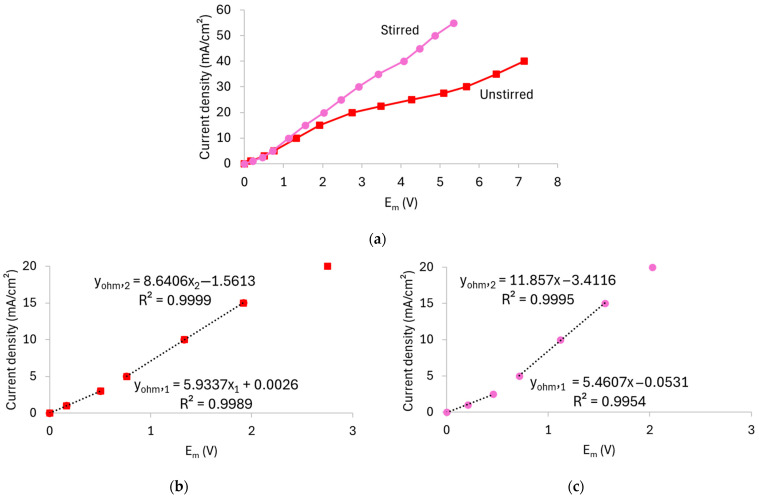
(**a**) Current–voltage curve for the AMV membrane in a 0.05 mol/L solution at pH 7, recorded under both unstirred and stirred conditions. Panels (**b**,**c**) show the magnified view of the ohmic regions of the unstirred and stirred systems, respectively.

Figure 4 shows the CVCs derived from the chronopotentiometric data, while Table 2 summarizes the values of the *i_lim_*, *R_ohm_*, and *R_overlim_*. To determine the *i_lim_*, Cowan–Brown curves were plotted, which are shown in Figure S3 of the Supplementary Material.

According to Figure 4a, the curve obtained without stirring displayed the typical shape with well-defined regions, while the curve with stirring showed a more linear behavior. In the latter case, the plateau separating the ohmic and overlimiting regions was significantly reduced, which made it more difficult to distinguish the regions. The two curves began to differ notably at current densities above the limiting current density of the unstirred system, as they were virtually superimposed in the ohmic region. This indicates that stirring has a pronounced effect on the membrane/solution system particularly under overlimiting conditions, as observed in the ChPs. In both cases, two distinct ohmic regions were observed, since the slope of the curves changed after approximately 3 mA/cm^2^ (see Figure 4b,c). This behavior can be attributed to the simultaneous transport of HPO_4_^2−^ and H_2_PO_4_^−^ species through the membrane, given their presence in the solution at pH 7 and pH 8 (Figure 2). As verified by Belashova et al. [32,35] and Rybalkina et al. [10,36], the first *R_ohm_* value corresponds to the transport of H_2_PO_4_^−^ ions. Upon entering the membrane, these ions are converted into HPO_4_^2−^, with the released protons migrating back toward the dilute-side interface. As the current density increases, HPO_4_^2−^ ions from the bulk solution become the dominant charge carriers, while protons continue to be released at the membrane–dilute interface. Consequently, the presence of protons at the membrane interface and the associated increase in the local conductivity explain the lower *R_ohm__2_* value compared to *R_ohm1_*. The first ohmic resistance (*R_ohm1_*) was comparable in both unstirred and stirred systems, whereas the second ohmic resistance (*R_ohm2_*) and mainly overlimiting resistance (*R_overlim_*) was lower in the stirred one due to the enhanced ion transport toward the membrane, highlighting the influence of hydrodynamic conditions on the transport of phosphate ions through the AMV membrane especially when operating at overlimiting current regimes.

The *i_lim_*, which marks the transition between the ohmic regime and the onset of intense concentration polarization, varied considerably with stirring from 15.9 to 33.9 mA/cm^2^, which can be attributed to the reduction of the diffusion boundary layer thickness.

These findings are consistent with the ChPs shown in Figure 3, which revealed that stirring induces intense E_m_ oscillations at high current densities. In this case, the pronounced oscillations in the ChPs and the shortening of the plateau length of the CVCs can be explained by the intensification of electroconvection under stirring, as verified in [37,38], since the stronger the electroconvection is, the shorter the plateau [39].

As stirring enhances phosphate ion transport and brings the system closer to a real electrodialysis system, the subsequent evaluations were conducted with solutions under stirring conditions.

### 3.2. Effect of pH Under Stirring Conditions

#### 3.2.1. AMV Membrane

**Figure 5:**
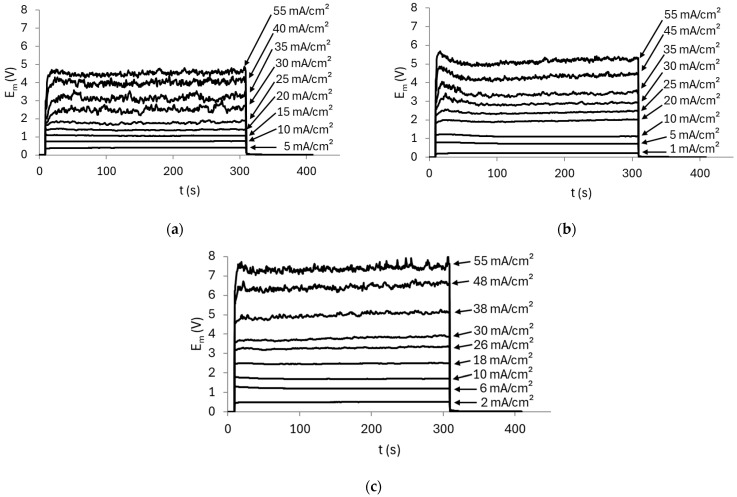
Chronopotentiometric curves obtained with the AMV membrane and solutions at pH (**a**) 4.4, (**b**) 7, and (**c**) 8 under stirring.

The effect of pH on the ion transfer through the homogeneous AMV membrane was evaluated using solutions at pH 4.4, 7, and 8. Figure 5 displays the chronopotentiometric data obtained in each of the three pH conditions.

**Figure 6:**
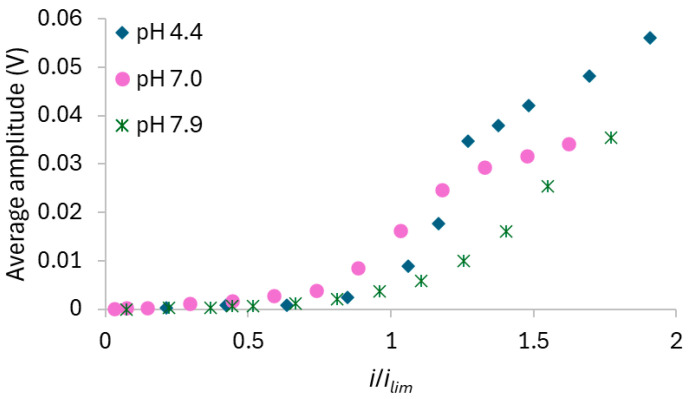
Amplitude of E_m_ recorded in the ChPs obtained at each current density using the AMV membrane and the three tested solutions.

The curves obtained at the lowest current densities exhibited similar shapes for all solutions, as expected, since the dominant mechanism was diffusion/migration, and in this case, E_m_ did not vary with time. In contrast, the curves obtained at higher current densities showed different behaviors for each solution, with intense oscillations in E_m_. The influence of initial pH on the intensity of the oscillations was assessed by calculating the average amplitude of E_m_ in the ChPs recorded at each current density using the three tested solutions. The corresponding results, plotted as a function of the ratio between the current density and the limiting current density of the membrane/solution system, are shown in Figure 6.

Figure 5 and Figure 6 show that no oscillations occurred with any solution at current densities up to around 20–30 mA/cm^2^ (*i*/*i_lim_* of approximately 1.0). Beyond this point, the oscillation amplitude increased significantly, particularly with the solution at pH 4.4 at the highest current densities, where values higher than 50 mV were reached. In contrast, the solution at an initial pH of 8 displayed the lowest oscillations intensity. Considering the predominant species in each solution at its initial condition (Figure 2), stronger oscillations were expected at pH 8 than at pH 4, since the conductivity of HPO_4_^2−^ is lower than that of H_2_PO_4_^−^, and lower ionic conductivity typically leads to more intense oscillations [37]. The opposite effect was observed for the solutions with the AMV membrane, likely due to water dissociation occurrence, which altered the predominant ionic species within the membrane/solution system. Table S1 in the Supplementary Material shows the pH values measured in the dilute compartment during chronopotentiometric experiments under different current densities. Notably, for the solution with an initial pH of 4.4, a pH decrease was observed upon current application, indicating water dissociation and the consequent accumulation of protons on the cathodic side of the membrane. In contrast, the pH of the solutions initially adjusted to pH 7 and 8 remained virtually unchanged under current. In these cases, intense water dissociation may still have occurred, but the pH was buffered by the simultaneous presence of H_2_PO_4_^−^ and HPO_4_^2−^ in the solution, which act as a buffer pair [7,40]. The occurrence of intense water dissociation in solutions at pH 7 and 8 is supported by Figure 6, which shows lower oscillation intensity in the ChPs, indicating reduced electroconvection. It is well known that electroconvection and water dissociation are competing phenomena [15]; therefore, the lower electroconvection observed in neutral and alkaline solutions suggests a strong occurrence of water dissociation.

As shown in Figure 5, the ChPs obtained with the solution at pH 7 exhibited a distinct behavior compared to the curves obtained with the other solutions. As discussed in Section 3.1.2., the emergence of a pronounced E_m_ peak suggests the occurrence of water dissociation at the membrane surface, followed by a shift in the chemical equilibrium at the membrane–solution interface. This was only observed with this solution due to the very similar initial concentrations of the H_2_PO_4_^−^ and HPO_4_^2−^ species.

It is worth mentioning that for both solutions at initial pH 7 and 8, the intense transport of generated OH^−^ ions through the membrane likely caused an increase in both the internal pH and the pH at the concentrated membrane surface, potentially leading to the formation of NH_3_, as illustrated in Figure 2. It is well established that NH_3_ formed at the enriched membrane surface tends to diffuse back into the dilute compartment, promoting water dissociation [41]. This did not occur with the pH 4.4 solution, since the low bulk pH prevented the membrane pH from reaching values around 9, at which the concentration of NH_3_ becomes significant. This led to an increase in membrane resistance, as evidenced by the higher E_m_ values observed in Figure 5 when comparing the curves obtained at the same current density for each solution.

**Figure 7:**
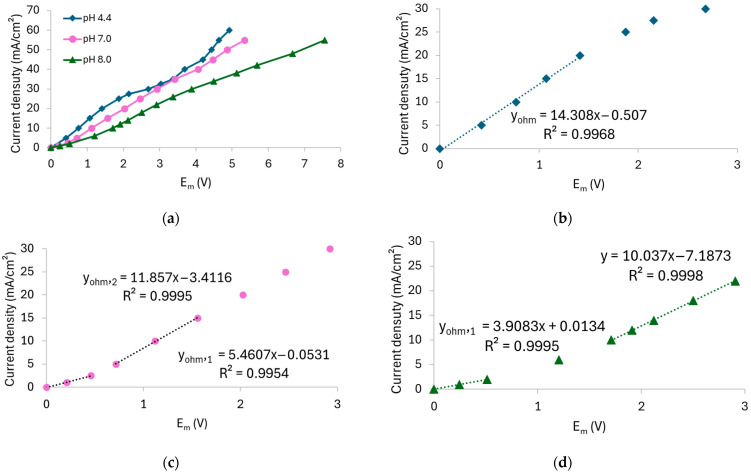
(**a**) Current–voltage curve for the AMV membrane in a 0.05 mol/L solution at different pH values under stirring. Figure (**b**–**d**) show the magnified view of the ohmic regions of the solutions at pH 4.4, 7, and 8, respectively.

From the chronopotentiometric data, the CVCs were obtained, which are shown in Figure 7, while the values of *i_lim_*, *R_ohm_*, and *R_overlim_* extracted from the CVCs and the *Q_eq_*^−^ calculated using Equation (5) are presented in Table 3. The Cowan–Brown plots used to determine the *i_lim_* are presented in Figure S4 of the Supplementary Material.

The CVC for the pH 4.4 solution exhibited the typical shape with three well-defined regions (Figure 7a,b). In contrast, the curves for pH 7 (Figure 7a,c) and pH 8 (Figure 7a,d) displayed an additional slope in the ohmic region due to equilibrium shifts and a significant reduction in the plateau length, as already discussed in Section 3.1.2. For the pH 7 solution, the transition between *R_ohm1_* and *R_ohm2_* was observed at 2.8 mA/cm^2^. However, for pH 8, it occurred at 1.8 mA/cm^2^, and this behavior was only observed for the neutral/alkaline solutions since both H_2_PO_4_^−^ and HPO_4_^2−^ species were present simultaneously at this condition. Similar behaviors have been reported for systems containing phosphate ions in different conditions [33,36] and other amphoteric solutions, such as EDTA [42], citric acid [43], and tartaric acid [43]. An increase in *R_ohm_* was observed with increasing pH, despite the rise in equivalent anionic charge. This can be explained by the predominance of H_2_PO_4_^−^ ions at pH 4.4 and HPO_4_^2−^ ions at pH 8, as the former exhibit higher diffusion coefficient (0.959·10^−^^5^ cm^2^/s) than the latter (0.759·10^−^^5^ cm^2^/s) [12]. Moreover, the acidic solution (pH 4.4) was more affected by H^+^ generation resulting from the shift in the phosphate equilibrium than the other solutions because, as previously discussed, the solutions at pH 7 and 8 exhibited buffering capacity, which prevented H^+^ accumulation in the solution. In this case, the protons generated at pH 4.4 increased the solution’s conductivity and consequently reduced its resistance, unlike the behavior observed for the pH 7 and 8 solutions.

Lastly, the limiting current density increased from the solution at pH 4.4 to 7, as expected, but unexpectedly decreased between pH 7 and 8. The *R_overlim_* increased with pH due to the occurrence of water dissociation under neutral/alkaline conditions, which led to the formation of NH_3_ and, consequently, increased resistance [41].

#### 3.2.2. HC-A Membrane

**Figure 8:**
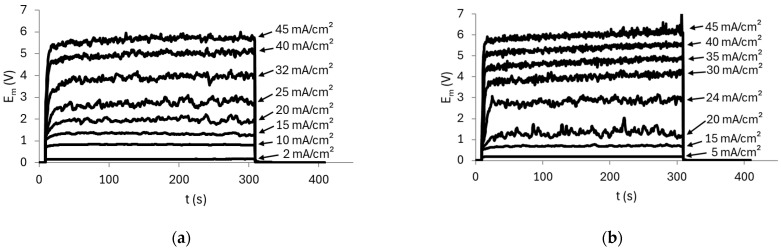
Chronopotentiometric curves obtained with the HC-A membrane and solutions at (**a**) pH 4.4 and (**b**) pH 8 under stirring.

The effect of pH on the heterogeneous HC-A membrane was evaluated using 0.05 mol/L diammonium phosphate solutions, under stirring, at initial pH values of 4.4 and 8, which are the lowest and highest pH values, respectively, evaluated with the AMV membrane. The ChPs obtained are shown in Figure 8.

**Figure 9:**
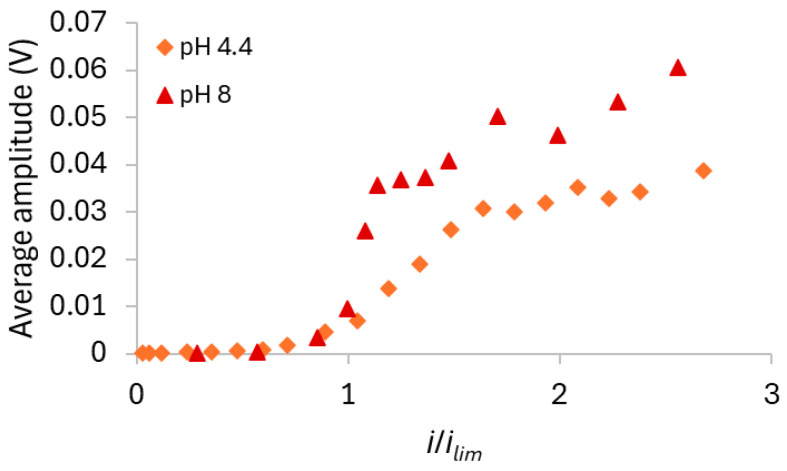
Amplitude of E_m_ recorded in the ChPs obtained at each current density using the HC-A membrane and the two tested solutions.

The curves at low current densities showed similar behaviors, with no inflection points, as expected. At high current densities, the curves exhibited the characteristic sharp increase in E_m_, indicative of severe concentration polarization, accompanied by pronounced oscillations. Figure 9 shows the average oscillation amplitude calculated for each system as a function of the ratio between the current density and the limiting current density of the membrane/solution system.

At low current densities, no oscillations were observed, as also shown in the ChPs of Figure 8. However, from approximately 17 mA/cm^2^ onward (*i*/*i_lim_* = 1), the oscillations increased markedly. By comparing Figure 6 and Figure 9, it can be observed that the membrane HC-A exhibited behavior opposite to that of the AMV membrane, as the HC-A showed much more intense oscillations under alkaline conditions (pH 8) than under acidic conditions (pH 4.4). This indicates that electroconvection was more pronounced at pH 8 with the HC-A membrane, likely due to the predominance of HPO_4_^2−^ ions, which have a higher hydration number than the H_2_PO_4_^−^ ions [44] predominant at acidic pH. As hydrated ions tend to enhance electroconvection by engaging larger volume of water in their movement [39,45], this could explain the greater oscillations intensity observed under these conditions. The relationship between pH and electroconvection intensity was the opposite for the homogeneous AMV membrane as this membrane favored intense water dissociation at high pH values, which in turn suppressed electroconvection. This is clearly shown in Figure S5, which presents a comparison of the average amplitude of the oscillations observed with the AMV and HC-A membranes at pH 4.4 and pH 8. The prevalence of the transport mechanisms in each membrane can also be observed from the pH values measured in the dilute compartment during the application of current pulses (Table S1 of the Supplementary Material), which are lower for the AMV membrane than for the HC-A membrane especially for the solution at initial pH of 4.4. Notably, electroconvection was significantly more intense with the AMV at pH 4.4 and with the HC-A at pH 8. The higher intensity of electroconvection with the HC-A at pH 8 was expected because of its structural heterogeneity, as the presence of poorly conductive regions promotes the development of tangential components of ion fluxes within the diffusion boundary layer, intensifying electroconvection [46].

According to Figure 8, neither of the two solutions exhibited the characteristic peak in E_m_ typically associated with water dissociation. This can be explained by the predominance of electroconvection with the HC-A membrane and by its heterogeneous structure and greater thickness (Table 1). Its structure consists of channels with a higher fraction of free electrolyte solution compared to the AMV membrane, which is thinner and denser. Therefore, Donnan exclusion tends to be less pronounced in the heterogeneous membrane, thereby reducing the intensity of the equilibrium shift effect of the phosphate species [47,48].

For the solution at pH 4.4, the curves at high current densities displayed an almost steady-state behavior. In contrast, the curves for the pH 8 solution showed a slight increase in E_m_ over time, which may be attributed to the formation of NH_3_ and the consequent increase in membrane resistance, similarly to what was observed with the AMV membrane. This interpretation is further supported by the higher E_m_ values observed at the same applied current densities (e.g., at 45 mA/cm^2^).

**Figure 10:**
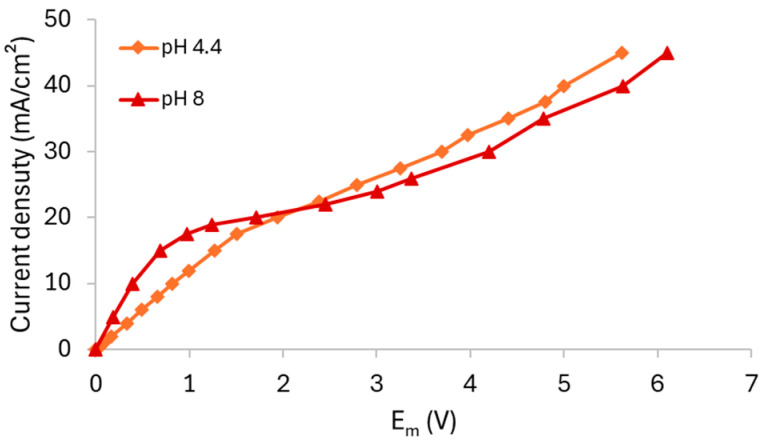
Current–voltage curve for the HC-A membrane in a 0.05 mol/L solution at different pH values under stirring.

Using the E_m_ data obtained from the chronopotentiometric tests, CVCs were constructed, as shown in Figure 10, while the values of *i_lim_*, *R_ohm_*, and *R_overlim_* extracted from the curves and *Q_eq_*^−^ calculated using Equation (5) are presented in Table 4.

Both curves exhibited three well-defined regions; however, notable differences were observed when compared to the curves obtained with the AMV membrane. For the HC-A membrane, only one inflection point related to the achievement of the limiting current density was observed. In this case, the curves did not clearly indicate the simultaneous transport of the species or any shift in the chemical equilibrium within the HC-A membrane, as observed in the ChPs. It is noteworthy that the observation of a single inflection point for the HC-A membrane may be related to the relatively high phosphate concentration used in this study. Rotta et al. [12], using electrochemical impedance spectroscopy, reported two inflection points in the ohmic region for the HC-A membrane only at lower phosphate concentrations (0.01 mol/L), which were not observed at higher concentrations (0.1 mol/L). This suggests that electrolyte concentration significantly influences transport behavior, particularly in heterogeneous membranes. The findings indicate that the AMV and HC-A membranes may exhibit distinct dependencies between solution concentration and the chemical equilibrium at the membrane–solution interface, likely due to differences in membrane morphology.

The electric resistances also showed different behavior compared to the AMV membrane. Notably, the *R_ohm_* of the solution at pH 8 was lower than that of the solution at pH 4.4, which can be explained by the greater equivalent anionic charge of the alkaline solution. Lastly, Table 4 shows that pH had an insignificant effect on *R_overlim_*, as it remained unchanged with increasing pH.

To facilitate the understanding and comparison of the results obtained in this study, Table 5 was constructed and summarizes the main findings. This approach provides a clearer visualization of the effects of the investigated variables on ion transport under the different experimental conditions.

## 4. Conclusions

In summary, the transport of phosphate ions through the homogeneous (AMV) and heterogeneous (HC-A) ion-exchange membranes was evaluated in an electrodialysis system using (NH_4_)_2_HPO_4_ 0.05 mol/L solutions under varying pH conditions. To better simulate real-world applications, the experiments were carried out under stirring.

Solution stirring altered the shape of the ChPs due to the appearance of intense oscillations in the membrane potential drop under current densities exceeding the limiting current density of the membrane/solution system. This behavior may be attributed to the intensified occurrence of electroconvection and the development of higher phosphate ion concentration gradients in both the bulk solution and the diffusion boundary layer. The current–voltage curves were also strongly influenced by stirring, showing a marked increase in the limiting current density, a reduction in electrical resistance under both ohmic and overlimiting regimes, and the absence of a clear distinction between these regions due to the shortening of the plateau region, which represents the transition between the two regimes.

Concerning the comparison of the membranes, under ohmic current conditions, the AMV membrane exhibited simultaneous transport of H_2_PO_4_^−^ and HPO_4_^2−^ species at pH 7 and 8, as revealed by chronopotentiometric and current–voltage analyses. This was not observed at pH 4, where H_2_PO_4_^−^ predominates. In contrast, no evidence of simultaneous transport of phosphate species was observed with the HC-A membrane under any pH condition.

Regarding mass transfer mechanisms under overlimiting current conditions, the AMV membrane promoted electroconvection at low pH and exhibited pronounced water dissociation at high pH, leading to local pH shifts and a consequent displacement of the chemical equilibrium within the membrane phase. Conversely, the HC-A membrane operated primarily under strong electroconvective regimes at both pH levels, particularly under alkaline conditions, without notable water dissociation or shifts in chemical equilibrium. In both cases, the curves indicated the formation of NH_3_ at the anodic surface of the membrane when operated at pH 7–8, which increased the membrane resistance. These findings highlight the distinct electrochemical behavior and pH sensitivity of the two membranes, which are critical factors to consider when selecting materials for efficient phosphate recovery using electrodialysis.

## Figures and Tables

**Table 1:** Key characteristics of Selemion AMV, Ionsep-HC-A, and Nafion 117 membranes.

	Selemion AMV	Ionsep-HC-A [19]	Nafion 117
Ion group attached	${-NR}_{3}^{+}$	${-NR}_{3}^{+}$	${-SO}_{3}^{-}$
Heterogeneity	Homogeneous [20]	Heterogeneous	Homogeneous [21,22]
Thickness (µm)	110–120 [23]	420 [24]	183 [25]
Ion-exchange capacity (Eq/kg)	1.68 eq/kg [20]	>1.8 (eq/kgdry)	1.86 eq/kg [26]
Density (g/cm^3^)	1.1 [27]	Not reported	1.98 [26]
Water uptake (%)	19 [23]	30–45	38 [25]
Resistance (Ω·cm^2^)	1.5–3.0 [23]	<20	Not reported
Transport number	>0.96 [20]	>0.89	Not reported

**Table 2:** *i_lim_*, *R_ohm_*, and *R_overlim_* obtained for the AMV membrane and a 0.05 mol/L solution at pH 7 for both unstirred and stirred conditions.

	*i_lim_* (mA/cm^2^)	*R_ohm,1_* (Ω·cm^2^)	*R_ohm,2_* (Ω·cm^2^)	*R_overlim_* (Ω·cm^2^)
Unstirred	15.9	168	116	148
Stirred	33.9	183	84	84

**Table 3:** *i_lim_*, *R_ohm_*, and *R_overlim_* obtained for the AMV membrane and a 0.05 mol/L solution at pH 4.4, 7, and 8 under stirring.

	$Q_{eq}^{-}$ (meq/L)	*i_lim_* (mA/cm^2^)	*R_ohm,1_* (Ω·cm^2^)	*R_ohm,2_* (Ω·cm^2^)	*R_overlim_* (Ω·cm^2^)
pH 4.4	49.8	23.6	Not present	70	48
pH 7	69.3	33.9	183	84	95
pH 8	93.2	27.1	255	99	145

**Table 4:** *i_lim_*, *R_ohm_*, and *R_overlim_* obtained for the HC-A membrane and a 0.05 mol/L solution at pH 4.4 and 8 under stirring.

	$Q_{eq}^{-}$ (meq/L)	*i_lim_* (mA/cm^2^)	*R_ohm_* (Ω·cm^2^)	*R_overlim_* (Ω·cm^2^)
pH 4.4	49.8	16.8	82	131
pH 8	93.2	17.6	39	132

**Table 5:** Summary of the main findings under different experimental conditions.

Category	AMV Membrane (Homogeneous)	HC-A Membrane (Heterogeneous)
Ion transport (ohmic conditions)	Simultaneous transport of H_2_PO_4_^−^ and HPO_4_^2−^ at pH 7 and 8. Only H_2_PO_4_^−^ at pH 4.	No evidence of simultaneous transport of different phosphate species at any pH.
Behavior under overlimiting current	Electroconvection at low pH. Significant water dissociation at high pH. Local pH shifts and chemical equilibrium displacement.	Strong electroconvection at all pH values. No water dissociation. No chemical equilibrium displacement.
Effect of solution stirring	Intense oscillations in chronopotentiograms under overlimiting current densities. Increase in limiting current density. Reduced electrical resistance. Compression of transition (plateau) region.	Same effects observed as in AMV membrane: changes in ChPs and CVCs due to stirring.
NH_3_ formation (pH 7–8)	Detected at the membrane–solution interface. Associated with increased membrane resistance.	Also detected at the membrane–solution interface. Also associated with increased membrane resistance.
pH sensitivity	High sensitivity: transport mechanisms and electrochemical behavior strongly influenced by pH.	Low sensitivity: consistent electrochemical behavior across different pH values.

## Data Availability

The original contributions presented in this study are included in the article. Further inquiries can be directed to the corresponding authors.

## Supplementary Materials

The following supporting information can be downloaded at: https://www.mdpi.com/article/10.3390/membranes15080230/s1, Figure S1: Photograph of the electrochemical cell. Figure S2: LSVs recorded over three consecutive scans for the AMV membrane in a 0.05 mol/L (NH_4_)_2_HPO_4_ solution at pH 7, under stirring. Figure S3: Cowan–Brown curves for the determination of the ilim of the AMV membrane in a 0.05 mol/L solution at pH 7, under (a) unstirred and (b) stirred conditions. Figure S4: Cowan–Brown curves for the determination of the *i_lim_* of the AMV membrane and solutions at pH (a) 4.4, (b) 7, and (c) 8 under stirring. Figure S5: Comparison of the average amplitude of the oscillations observed with the AMV and HC-A membranes at (a) pH 4.4 and (b) pH 8. Table S1: pH values of the dilute solution measured during the chronopotentiometric experiments under different current densities.
