# Supplementary material for: Phosphate Transport Through Homogeneous and Heterogeneous Anion-Exchange Membranes: A Chronopotentiometric Study for Electrodialytic Applications
Source: Membranes (Basel). 2025 Jul 31;15(8):230. doi: 10.3390/membranes15080230 (PMC12388024; doi:10.3390/membranes15080230)
Supplement: Supplementary file 1 [file membranes-15-00230-s001.zip › membranes-3762997-supplementary.pdf]

Phosphate transport through homogeneous and heterogeneous anion-exchange membranes: A chronopotentiometric study for electrodialytic applications

SUPPLEMENTARY MATERIAL

Kayo Santana-Barros [1*], Manuel César Martí-Calatayud [1*], Svetlozar Velizarov [2], Valentín Pérez-Herranz [1]

1. IEC Group, ISIRYM, Universitat Politècnica de València – Spain. Address: Camí de Vera s/n, 46022, P.O. Box 22012, València E-46071, Spain.
2. LAQV / REQUIMTE, Department of Chemistry, NOVA School of Science and Technology, NOVA FCT, Universidade NOVA de Lisboa, 2829-516 Caparica, Portugal.

*Correspondence: K.S.B: kasanbar@alumni.upv.es ; M.C.M.C: mcmarti@iqn.upv.es

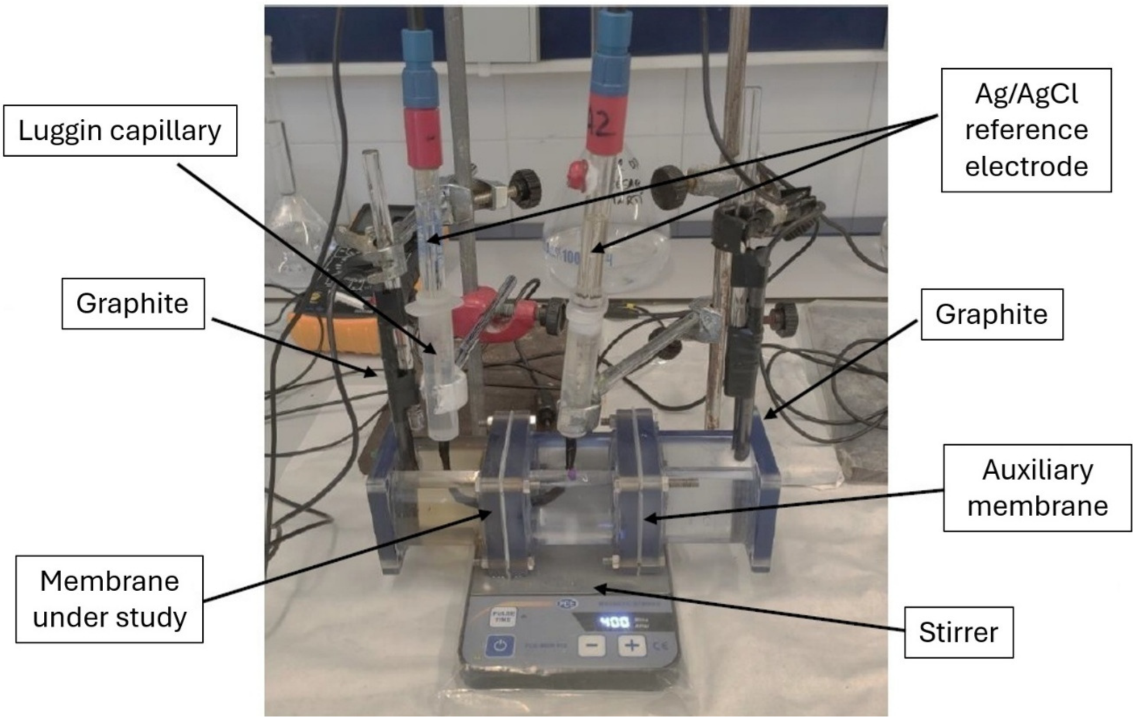

Figure S1. Photograph of the electrochemical cell.

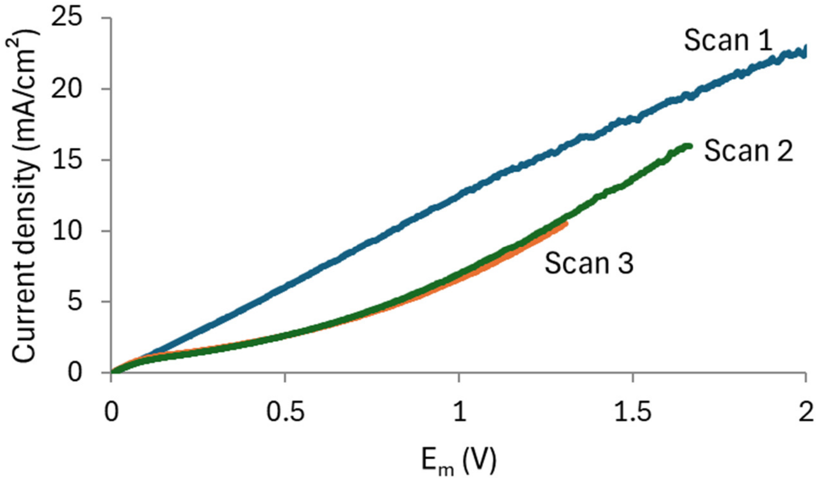

Figure S2. LSVs recorded over three consecutive scans for the AMV membrane in a 0.05 mol/L $(NH_4)_2HPO_4$ solution at pH 7, under stirring.

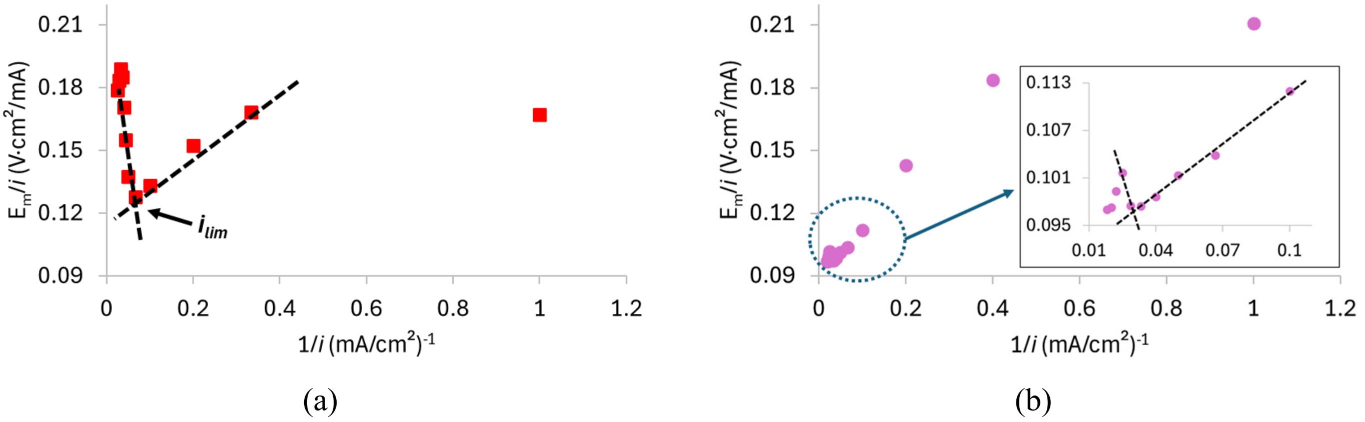

Figure S3. Cowan–Brown curves for the determination of the i_{lim} of the AMV membrane in a 0.05 mol/L solution at pH 7, under (a) unstirred and (b) stirred conditions.

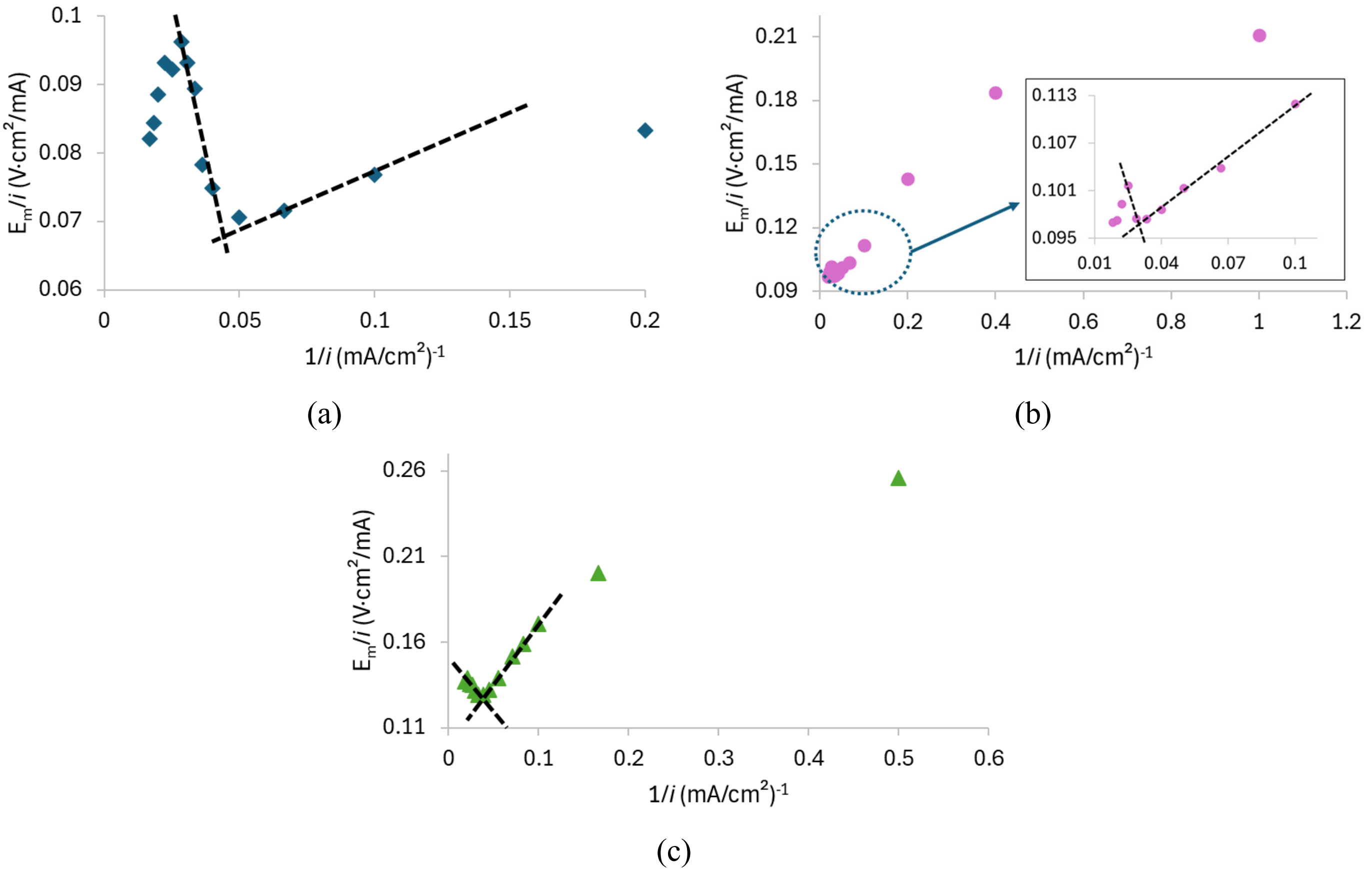

Figure S4. Cowan–Brown curves for the determination of the i_{lim} of the AMV membrane and solutions at pH (a) 4.4, (b) 7, and (c) 8 under stirring.

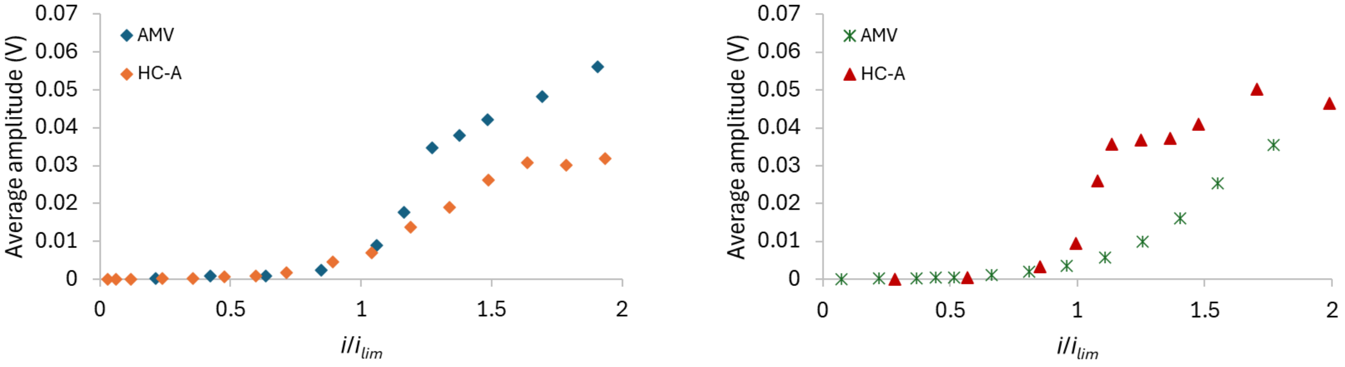

(a) (b)

Figure S5. Comparison of the average amplitude of the oscillations observed with the AMV and HC-A membranes at (a) pH 4.4 and (b) pH 8.

Table S1. pH values of the dilute solution measured during the chronopotentiometric experiments under different current densities.

AMV membrane		HC-A membrane	
Initial solution pH: 4.4			
Current density (mA/cm²)	pH	Current density (mA/cm²)	pH
30	4.05	32	4.25
45	3.64	45	3.84
Initial solution pH: 7.0			
Current density (mA/cm²)	pH		
20	7.01		
35	7.04		
45	7.01		
55	7.03		
Initial solution pH: 8.0			
Current density (mA/cm²)	pH	Current density (mA/cm²)	pH
12	7.97	20	7.98
34	7.91	26	8.04
45	7.90	40	8.00
55	7.86	45	7.97
